# Preventive ceftriaxone in patients at high risk of stroke-associated pneumonia. A post-hoc analysis of the PASS trial

**DOI:** 10.1371/journal.pone.0279700

**Published:** 2022-12-30

**Authors:** Wouter M. Sluis, Willeke F. Westendorp, Diederik van de Beek, Paul J. Nederkoorn, H. Bart van der Worp

**Affiliations:** 1 Department of Neurology and Neurosurgery, UMC Utrecht Brain Center, University Medical Center Utrecht, Utrecht University, Utrecht, The Netherlands; 2 Department of Neurology, Amsterdam Neuroscience, Amsterdam UMC location University of Amsterdam, Amsterdam, The Netherlands; Bach Mai Hospital, VIET NAM

## Abstract

**Background:**

Infections complicate the acute phase of stroke in one third of patients and especially pneumonia is associated with increased risk of death or dependency. In randomized trials of stroke patients, preventive antibiotics reduced overall infections, but did not reduce pneumonia or improve outcome. This may be explained by broad selection criteria, including many patients with a low risk of pneumonia. To assess the potential of selection of patients at high risk of pneumonia, we performed a post-hoc analysis in the Preventive Antibiotics in Stroke Study (PASS).

**Methods:**

PASS was a multicentre phase 3 trial in acute stroke patients who were randomized to preventive ceftriaxone for four days within 24 hours or standard care. For this analysis patients were divided based on the ISAN risk score for pneumonia as follows: low (0–6), medium (7–14) and high (15–21). Primary outcomes were pneumonia rate during admission as judged by the treating physician, and by an independent committee; secondary outcomes were overall infections and unfavorable outcome (modified Rankin Scale ≥3). We adjusted with multivariable regression for possible confounders: age, stroke subtype and severity, pre-stroke dependency and diabetes.

**Results:**

Pneumonia occurred more frequently in higher risk groups (25.7% (high), 9.0% (medium) 1.5%, (low)). The absolute difference in pneumonia rate between patients treated with ceftriaxone or standard care increased with the ISAN score (low: 0.5%, medium: 1.2%, high: 10.1%). After adjustment ceftriaxone reduced overall infections in the low and medium groups, not in the high-risk group. There was a trend towards reduction of pneumonia as judged by the committee (3.7% vs 13.6%, aOR = 0.164, p = 0.063) in the high-risk group.

**Conclusions:**

This post-hoc analysis of PASS confirmed higher rates of pneumonia with higher ISAN scores, and suggests that in acute stroke patients with an ISAN score of ≥15, preventive ceftriaxone for four days may reduce pneumonia rate.

## Introduction

Infections complicate the acute phase of stroke in about one third of patients and are associated with an increased risk of death or dependency [1]. This association is stronger for pneumonia than for urinary tract infections [1]. Important predictors for pneumonia after stroke are a higher age, dysphagia and stroke severity [2]. In randomized trials of patients with acute stroke, preventive antibiotics reduced the overall infection rate, but did not reduce the rate of pneumonia or improve functional outcome [3]. This may be explained in part by broad selection criteria for patients in previous trials, including many patients with a low risk of pneumonia.

The ongoing randomized PREvention of Complications to Improve Outcome in elderly patients with acute Stroke (PRECIOUS) trial, recruits only patients aged 66 years or older with moderately-severe to severe stroke. Patients are randomized to preventive treatment with ceftriaxone, metoclopramide, paracetamol, any combination of these, or to standard care alone [4].

To assess the potential of ceftriaxone to prevent pneumonia in these patients, we performed a post-hoc analysis of patients with a high risk of pneumonia based on the integer-based pneumonia risk score (ISAN) [5] in the Preventive Antibiotics in Stroke Study (PASS) [6].

## Methods

We performed a post-hoc analysis of PASS (ISRCTN66140176), of which the methods and results have been reported previously [6]. In short, PASS was a multicentre, randomized, open-label, clinical trial with blinded outcome assessment of preventive ceftriaxone in patients with acute stroke. Patients were randomly assigned in a 1:1 ratio to treatment with ceftriaxone 2 g once daily for 4 days in addition to stroke unit care or to stroke unit care alone. The institutional review board of the Academic Medical Center (Amsterdam, Netherlands) approved the study protocol.

In PASS, patients were eligible for inclusion if they were aged 18 years or older and had ischemic stroke or intracerebral hemorrhage in the previous 24 hours with a score of 1 or more on the National Institutes of Health Stroke Scale (NIHSS). Exclusion criteria were clinical signs of infection on hospital admission requiring antibiotic therapy, use of antibiotics less than 24 hours before admission, pregnancy, hypersensitivity for any cephalosporin, previous anaphylaxis for penicillin derivatives, and imminent death on hospital admission. All patients or their legal representatives provided written informed consent. For the current study, baseline demographics, comorbidities, stroke type and data on dysphagia assessment were collected. In the Case Record Form of PASS researchers were asked to provide details on whether a swallow screening was performed and the results of the screening. Dysphagia was scored as present when patients had an abnormal swallow test, as absentin case of a normal swallow test or if no swallow test was performed because of excellent recovery.

For the present post-hoc analysis, patients were categorised in subgroups based on the ISAN risk score [5]. The ISAN risk score has been developed in a population of patients with either ischemic stroke or intracerebral hemorrhage and all elements of the risk score are available in PASS. Pre-stroke functional dependency was defined as a modified Rankin Scale (mRS) score of 3 or higher before stroke. The components and weightings of the ISAN score are shown in **Table 1.** To investigate the potential of preventive ceftriaxone to reduce the occurrence of pneumonia in the PRECIOUS population we divided the ISAN score as follows: low (0–6), medium (7–14) or high risk (15–21). Patients recruited into PRECIOUS will by definition have a ISAN score of 7 or higher and fall in to the medium- or high-risk group. A sensitivity analysis with the original 4 categories (low 0–5; medium 6–10; high 11–14; very high 15+) will be performed.

**Table 1:** Components and weightings of the ISAN score [4].

Item	Score
*Age in years*	
<60	0
60–69	3
70–79	4
80–89	6
90+	8
*Sex*	
Female	0
Male	1
*NIHSS on admission*	
0 to 4	0
5 to 15	4
16 to 20	8
21+	10
*Functional dependency before stroke*	
Independent	0
Not independent	2

NIHSS indicates National Institutes of Health Stroke Scale.

The current study has a co-primary outcome: pneumonia rate (as assessed by the treating physician) and pneumonia rate as assessed by an independent adjudication committee (masked to treatment allocation). Secondary outcomes were infection rate (as assessed by the treating physician and the adjudication committee) and an unfavorable outcome at 90 ± 14 days, defined as a score on the mRS of 3 or higher. Infections were categorised as diagnosed by the clinician, and as judged by an independent adjudication committee (masked to treatment allocation) according to modified Centers for Disease Control and Prevention criteria [7]. To assess the effects of ceftriaxone we used multivariate logistic regression and adjusted for factors known to influence pneumonia rate or functional outcome: age, NIHSS score, type of stroke, pre-stroke mRS, and diabetes mellitus. Statistical significance was defined as a p value <0.05. All statistical analyses were performed using SPSS software version 26.0 (IBM Corp., Armonk, NY, USA).

## Results

We used data from all 2538 patients included in the intention-to-treat analysis of PASS [5]. The mean age of the patients was 71 years (SD 12.7) and the median NIHSS score 5 (IQR 3–9). Of all patients, 2125 (84%) had ischemic stroke, 269 (11%) intracerebral hemorrhage, 93 (4%) a transient ischemic attack, and 51 (2%) a stroke mimic. Other baseline characteristics of PASS are described elsewhere [6]. One thousand one hundred sixty-six patients (46%) had a low ISAN score, 1259 (50%) a medium score, and 113 (4.5%) a high score. Baseline characteristics per treatment stratum are summarised in **Table 2** for each ISAN group. Patients randomized to ceftriaxone or standard care were comparable for age, sex, comorbidities, pre-stroke mRS, stroke severity, stroke subtype and dysphagia in the low, medium and high ISAN groups. In the group of patients who were randomized to standard care, 193 patients eventually received antibiotics (15.2%). The proportion of patients with standard care in the higher ISAN group was 44.1% (n = 26).

**Table 2:** Baseline characteristics of patients with a low, medium or high ISAN score stratified by treatment group.

	Low ISAN score		Medium ISAN score		High ISAN score	
	Ceftriaxone	Standard care	Ceftriaxone	Standard care	Ceftriaxone	Standard care
	n = 581	n = 585	n = 633	n = 626	n = 54	n = 59
Age (mean, SD)	64.7 (12.2)	64.8 (12.4)	76.2 (9.8)	76.7 (10.2)	83.9 (7.9)	84.4 (7.0)
Male sex	328 (56.5)	338 (57.8)	361 (57.0)	359 (57.3)	30 (55.6)	28 (47.5)
Pre-stroke mRS (median, IQR)	0 (0–0)	0 (0–0)	0 (0–2)	0 (0–2)	2 (0–3)	1 (0–3)
*Comorbidities*						
Atrial fibrillation	55 (9.5)	63 (10.8)	113 (17.9)	122 (19.5)	16 (29.6)	22 (37.3)
Previous stroke	168 (28.9)	178 (30.4)	215 (34.1)	227 (36.3)	23 (42.6)	16 (27.1)
Hypercholesterolaemia	151 (26.0)	158 (27.1)	165 (26.4)	165 (26.7)	16 (29.6)	10 (17.2)
Hypertension	298 (51.3)	296 (50.7)	361 (57.2)	371 (59.4)	35 (64.8)	39 (66.1)
Myocardial infarction	61 (10.5)	52 (8.9)	103 (16.3)	103 (16.5)	8 (14.8)	4 (6.8)
Cardiac valve disease	34 (5.9)	30 (5.1)	55 (8.7)	40 (6.4)	6 (11.1)	8 (13.6)
Peripheral vascular disease	33 (5.7)	40 (6.8)	53 (8.4)	56 (9.0)	5 (9.3)	3 (5.2)
COPD	47 (8.1)	39 (6.7)	64 (10.1)	49 (7.9)	4 (7.4)	5 (8.5)
Diabetes mellitus	111 (19.1)	108 (18.5)	130 (20.6)	131 (20.9)	10 (18.5)	12 (20.3)
Alcoholism	34 (5.9)	34 (5.8)	22 (3.5)	28 (4.5)	2 (3.8)	1 (1.7)
Malignancy	41 (7.1)	41 (7.0)	65 (10.3)	75 (12.0)	6 (11.1)	5 (10.3)
Immunocompromised	181 (31.2)	174 (29.7)	208 (32.9)	217 (34.7)	16 (29.6)	18 (30.5)
Current smoker	195 (33.7)	181 (30.9)	120 (19.3)	118 (19.1)	4 (7.8)	2 (3.7)
NIHSS (median, IQR)	3.0 (2.0–4.0)	3.0 (2.0–4.0)	7.0 (5.0–12.0)	7.0 (5.0–12.0)	19.5 (17.0–22.3)	21.0 (18.0–22.0)
*Stroke type*						
Ischemic stroke	481 (82.8)	478 (81.7)	534 (84.4)	539 (86.1)	43 (79.6)	50 (84.7)
Intracerebral hemorrhage	51 (8.8)	53 (9.1)	82 (13.0)	65 (10.4)	10 (18.5)	8 (13.6)
Transient ischemic attack	33 (5.7)	33 (5.6)	10 (1.6)	16 (2.6)	1 (1.9)	0 (0.0)
Other	16 (2.8)	21 (3.6)	7 (1.1)	6 (1.0)	0 (0.0)	1 (1.7)
Dysphagia	56 (10.1)	47 (8.4)	215 (37.4)	227 (38.7)	36 (78.3)	42 (89.4)

SD indicates standard deviation; mRS = modified Rankin Scale; IQR, interquartile range; COPD, chronic obstructive pulmonary disease; NIHSS National Institutes of Health Stroke Scale.

**Fig 1:**
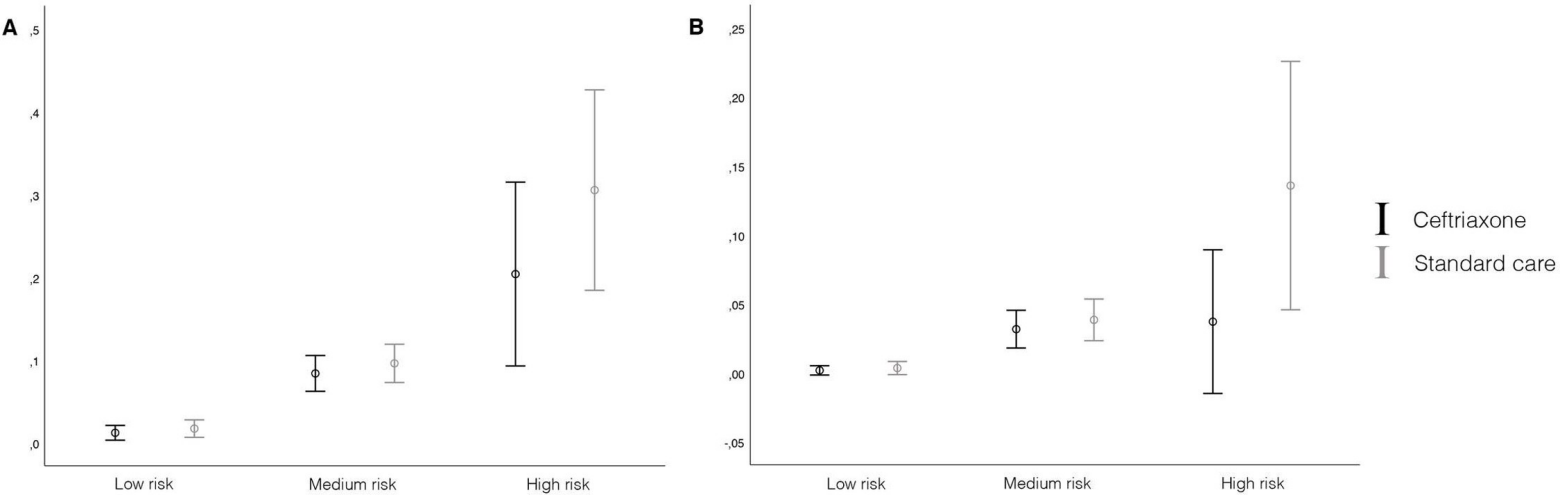
2-panel figure showing pneumonia rate as judged by the physician (panel A) and by the adjudication panel (panel B) per treatment stratum for low, medium and high ISAN-score.

In the overall population, 159 patients (6.3%) had a pneumonia as diagnosed by the treating physician and 57 (2.2%) as diagnosed by the adjudication panel. Pneumonia was more frequently diagnosed in higher risk groups (low: 17/1166 (1.5%); medium: 13/1259 (9.0%); high 29/113 (25.7%)) and the difference in pneumonia incidence as judged by the physician between patients who were randomized to ceftriaxone or standard care was greater in the highest ISAN group in favour of ceftriaxone (absolute risk reduction 0.5% (95% CI -0.9 to 1.9) for low risk, 1.2% (95% CI, -1.9 to 4.4) for medium risk, and 10.1% (95% CI, -5.8 to 26.1) for high risk). This was comparable for pneumonia incidence as judged by the adjudication panel (0.1% (95% CI, -0.4 to 0.8) for low, 0.6% (95%CI, -1.4 to 2.7) for medium and 9.9% (95% CI, -0.2 to 19.9) for high risk) (**Fig 1)**. After adjustment for confounders there was a reduction in the rate of pneumonia as judged by the adjudication panel in patients with a high ISAN score randomized to ceftriaxone (2/54 (3.7%)) as compared to standard care (8/59 (13.6%) aOR 0.164, p = 0.063), this was not statistically significant. This was not seen in patients with a low or medium ISAN score or in pneumonia incidence as judged by the treating physician. (**Table 3)**. In patients randomized to ceftriaxone there was a lower overall infection rate in the low and medium ISAN categories as judged by the physician and the adjudication panel (**Table 3)**. The proportion of patients with an unfavorable outcome increased per ISAN group, but was comparable between patients randomized to ceftriaxone and standard care. Results of the sensitivity analysis with the original four categories can be found in the supplementary **S1 Table.**

**Table 3:** Univariate and multivariate analysis of outcomes per ISAN score group.

	Ceftriaxone	Standard care	OR (95% CI)	p-value
**Low ISAN-score**				
Pneumonia physician	1.2 (7/581)	1.7 (10/585)	0.701 (0.27–1.86)	0.475
			0.674 (0.25–1.83)*	0.439
Pneumonia panel	0.2 (1/581)	0.3 (2/585)	0.503 (0.05–5.56)	0.575
			0.550 (0.05–6.21)*	0.628
Infection physician	3.4 (20/581)	6.3 (37/585)	0.528 (0.30–0.92)	**0.025**
			0.500 (0.28–0.88)*	**0.020**
Infection panel	0.3 (2/581)	2.7 (16/585))	0.123 (0.03–0.54)	**0.005**
			0.125 (0.03–0.55)*	**0.006**
Unfavorable outcome	17.9 (103/575)	17.4 (101/580)	1.035 (0.77–1.40)	0.824
			1.037 (0.75–1.43)*	0.825
**Medium ISAN-score**				
Pneumonia physician	8.4 (53/633)	9.6 (60/626)	0.862 (0.59–1.27)	0.452
			0.860 (0.58–1.29)*	0.465
Pneumonia panel	3.2 (20/633)	3.8 (24/626)	0.818 (0.45–1.50)	0.515
			0.843 (0.45–1.57)*	0.590
Infection physician	14.7 (93/633)	24.0 (150/626)	0.547 (0.41–0.73)	**<0.001**
			0.526 (0.39–0.71)*	**<0.001**
Infection panel	5.1 (32/633)	9.6 (60/626)	0.502 (0.32–0.78)	**0.002**
			0.483 (0.31–0.77)*	**0.002**
Unfavorable outcome	52.5 (220/628)	56.1 (347/618)	0.865 (0.69–1.08)	0.202
			0.819 (0.64–1.06)*	0.122
**High ISAN-score**				
Pneumonia physician	20.4 (11/54)	30.5 (18/59)	0.583 (0.25–1.38)	0.220
			0.684 (0.26–1.79)*	0.437
Pneumonia panel	3.7 (2/54)	13.6 (8/59)	0.245 (0.05–1.21)	0.084
			0.164 (0.02–1.10)*	0.063
Infection physician	31.5 (17/54)	52.5 (31/59)	0.415 (0.19–0.90)	**0.025**
			0.493 (0.21–1.16)*	0.104
Infection panel	11.1 (6/54)	22.0 (13/59)	0.442 (0.16–1.26)	0.127
			0.482 (0.16–1.49)*	0.204
Unfavorable outcome	94.4 (52/54)	91.5 (54/59)	1.57 (0.36–6.93)	0.548
			1.10 (0.13–9.28)*	0.933

* corrected for age, NIHSS, type of stroke, mRS at baseline, diabetes mellitus

## Discussion

This post-hoc subgroup analysis of PASS confirmed higher rates of pneumonia with higher ISAN scores, and also suggests that in patients with acute stroke and an ISAN score of 15 or higher, preventive ceftriaxone for four days may reduce the risk of pneumonia. Unfortunately, just 113 patients had such a high score and analyses of this subgroup are therefore likely underpowered.

Whereas stroke severity in general was low in PASS (median NIHSS 5 in both arms), causing a generally low ISAN score, median NIHSS scores of patients in the active treatment arms of other randomized trials of preventive antibiotics ranged from 14 to 17 [8–11]. In PANTHERIS and MISS a reduction in the rate of any infection was seen, but no significant reduction in the rate of pneumonia and there was no effect on functional outcome [8, 9]. Both trials had a small sample size and were probably underpowered to detect differences in outcome. ESPIAS was stopped early because of futility [10]. STROKE-INF was the only larger trial that included patients with a higher stroke severity, and showed a reduction in the rate of any infection with preventive antibiotics, but not in stroke-associated pneumonia or functional outcome [11]. In an individual patient data meta-analysis of patients in PASS and STROKE-INF, subgroup analyses of patients with ISAN scores divided in two and three groups according to pneumonia risk did not influence treatment response of preventive antibiotics [12]. Possible explanations for the lack of effect could be the late start of treatment in STROKE-INF (up to 48h of stroke onset) and the fact that a considerable amount of patients in the control group of STROKE-INF also received an antibiotic.

Not surprisingly, patients with the highest ISAN score not only had the highest rate of pneumonia, but also the greatest risk of a poor functional outcome. The higher age and higher NIHSS scores which drive the ISAN score are also established predictors of a poor functional outcome and death [13–15]. In none of our ISAN subgroups ceftriaxone had an effect on the risk of a poor functional outcome, but especially in the highest subgroup 95% confidence intervals were very wide and a clinically relevant effect cannot be excluded.

Given the apparent large absolute reduction in the risk of pneumonia with ceftriaxone in the subgroup of patients with a high ISAN score, an evaluation in a larger population of patients with acute stroke and a high ISAN score is warranted. The ongoing PRECIOUS trial currently assesses whether prevention of aspiration, infection, or fever with metoclopramide, ceftriaxone, paracetamol, or any combination of these in the first 4 days after stroke onset can prevent these complications and improve outcome in elderly patients with moderately severe to severe stroke [4]. The planned large sample size together with the inclusion limited to elderly patients with moderately severe to severe stroke (only patients with an ISAN score of 7 or higher will be included in PRECIOUS) could confirm or refute the observed trend in this post-hoc analysis. If ceftriaxone reduces the risk of pneumonia in PRECIOUS, it can also be assessed whether this translates in improved functional outcomes in these patients.

## Conclusions

This post-hoc analysis of PASS confirmed higher rates of pneumonia with higher ISAN scores and suggests that patients with acute stroke who are at a high risk of stroke-associated pneumonia are a worthwhile target for studies on preventive antibiotics.

## Supporting information

S1 Table Sensitivity univariate and multivariate analysis with the original ISAN categories. (DOCX)
