# Supplementary material for: Preventive ceftriaxone in patients at high risk of stroke-associated pneumonia. A post-hoc analysis of the PASS trial
Source: PLoS One. 2022 Dec 30;17(12):e0279700. doi: 10.1371/journal.pone.0279700 (PMC9803205; doi:10.1371/journal.pone.0279700)
Supplement: S1 Table — (DOCX) [file pone.0279700.s001.docx]

**Supplemental Table S1**

|  | Ceftriaxone | Standard care | OR (95% CI) |  |
| --- | --- | --- | --- | --- |
| Low ISAN-score (0-5) | | | | |
| Pneumonia physician | 1.4 (7/503) | 1.6 (8/496) | 0.86 (0.31-2.39)  0.81 (0.29-2.32)* |  |
| Pneumonia panel | 0.2 (1/503) | 0.2 (1/496) | 0.99 (0.06-15.81)  0.96 (0.06-15.75)* |  |
| Infection physician | 3.6 (18/503) | 6.0 (30/496) | 0.58 (0.32-1.05)  0.533 (0.29-0.99)* |  |
| Infection panel | 0.4 (2/503) | 2.6 (13/496) | 0.15 (0.03-0.66)  0.15 (0.03-0.67)* |  |
| Unfavourable outcome | 15.1 (75/497) | 14.4 (71/493) | 1.06 (0.74-1.50)  1.03 (0.71-1.50)* |  |

* corrected for age, NIHSS, type of stroke, mRS at baseline, diabetes mellitus

|  | Ceftriaxone | Standard care | OR (95% CI) |  |
| --- | --- | --- | --- | --- |
| Medium ISAN-score (6-10) | | | | |
| Pneumonia physician | 4.1 (21/508) | 6.5 (33/510) | 0.62 (0.36-1.09)  0.62 (0.35-1.12)* |  |
| Pneumonia panel | 1.6 (8/508) | 2.9 (15/510) | 0.53 (0.22-1.26)  0.57 (0.23-1.38)* |  |
| Infection physician | 8.5 (43/508) | 17.8 (91/510) | 0.43 (0.29-0.63)  0.40 (0.27-0.60)* |  |
| Infection panel | 3.0 (15/508) | 7.1 (36/510) | 0.40 (0.22-0.74)  0.40 (0.21-0.74)* |  |
| Unfavourable outcome | 41.3 (210/508) | 44.7 (228/510) | 0.86 (0.67-1.10)  0.80 (0.61-1.05)* |  |

* corrected for age, NIHSS, type of stroke, mRS at baseline, diabetes mellitus

|  | Ceftriaxone | Standard care | OR (95% CI) |  |
| --- | --- | --- | --- | --- |
| High ISAN-score (11-14) | | | | |
| Pneumonia physician | 15.8 (32/203) | 14.1 (29/205) | 1.14 (0.66-1.96)  1.16 (0.66-2.05)* |  |
| Pneumonia panel | 5.9 (12/203) | 4.9 (10/205) | 1.23 (0.52-2.90)  1.34 (0.55-3.26)* |  |
| Infection physician | 25.6 (52/203) | 32.2 (66/205) | 0.73 (0.47-1.11)  0.73 (0.46-1.15)* |  |
| Infection panel | 8.4 (17/203) | 13.2 (27/205) | 0.60 (0.32-1.14)  0.58 (0.30-1.13)* |  |
| Unfavourable outcome | 73.3 (148/202) | 73.4 (149/203) | 1.01 (0.65-1.57)  0.97 (0.60-1.57)* |  |

* corrected for age, NIHSS, type of stroke, mRS at baseline, diabetes mellitus

|  | Ceftriaxone | Standard care | OR (95% CI) |  |
| --- | --- | --- | --- | --- |
| Very High ISAN-score (15-21) | | | | |
| Pneumonia physician | 20.4 (11/54) | 30.5 (18/59) | 0.58 (0.25-1.38)  0.68 (0.26-1.79)* |  |
| Pneumonia panel | 3.7 (2/54) | 13.6 (8/59) | 0.25 (0.05-1.21)  0.16 (0.02-1.10)* |  |
| Infection physician | 31.5 (17/54) | 52.5 (31/59) | 0.42 (0.19-0.90)  0.49 (0.21-1.16)* |  |
| Infection panel | 11.1 (6/54) | 22.0 (13/59) | 0.44 (0.16-1.26)  0.48 (0.16-1.49)* |  |
| Unfavourable outcome | 94.4 (52/54) | 91.5 (54/59) | 1.57 (0.36-6.93)  1.10 (0.13-9.28)* |  |

* corrected for age, NIHSS, type of stroke, mRS at baseline, diabetes mellitus
